# A Rare Penetrating Injury to an Aberrant Right Subclavian Artery and the Turbulent Course of Endovascular Repair

**DOI:** 10.7759/cureus.87848

**Published:** 2025-07-13

**Authors:** Sai Swarupa Vulasala, Aryan Sharma, Dheeraj Gopireddy, Steven Morales-Rivera, Grit Adler

**Affiliations:** 1 Diagnostic Radiology, University of Florida College of Medicine – Jacksonville, Jacksonville, USA; 2 Biology, Creekside High School, St. Johns, USA; 3 Interventional Radiology, University of Florida College of Medicine – Jacksonville, Jacksonville, USA

**Keywords:** aberrant right subclavian artery, aberrant subclavian, catheter-directed thrombolysis, endovascular interventions, esophageal-arterial fistula, penetrating trauma neck

## Abstract

Penetrating neck trauma carries high morbidity and mortality due to the involvement of major vascular structures and anatomical complexity. A penetrating injury to an aberrant right subclavian artery (ARSA) is very rare and presents unique management challenges. This case details a 27-year-old male patient with a gunshot wound which resulted in esophageal injury and ARSA pseudoaneurysm. Following endovascular repair of the ARSA pseudoaneurysm, the clinical course was complicated by several events, some related to the stents and others unrelated to them.

## Introduction

Aberrant right subclavian artery (ARSA), also known as a lusoria artery, is a rare anatomic variant where the vessel originates from the left-sided aortic arch distal to the left subclavian artery. Traditionally, the right subclavian artery arises from the right brachiocephalic trunk. ARSA occurs in only 0.4-2% of the population [1], and often takes a retroesophageal course. Although usually an incidental finding, approximately 10% of patients with ARSA experience dysphagia due to esophageal compression, known as dysphagia lusoria [2].

Penetrating neck injuries have a high risk of injury to critical structures, such as the vasculature, lung apex, esophagus, brachial plexus, and trachea. In this case report, we discuss a penetrating neck injury involving both the esophagus and ARSA, treated via surgical and endovascular approaches. We primarily focus on the series of complications from the endovascular intervention in our patient. 

## Case presentation

**Figure 1:**
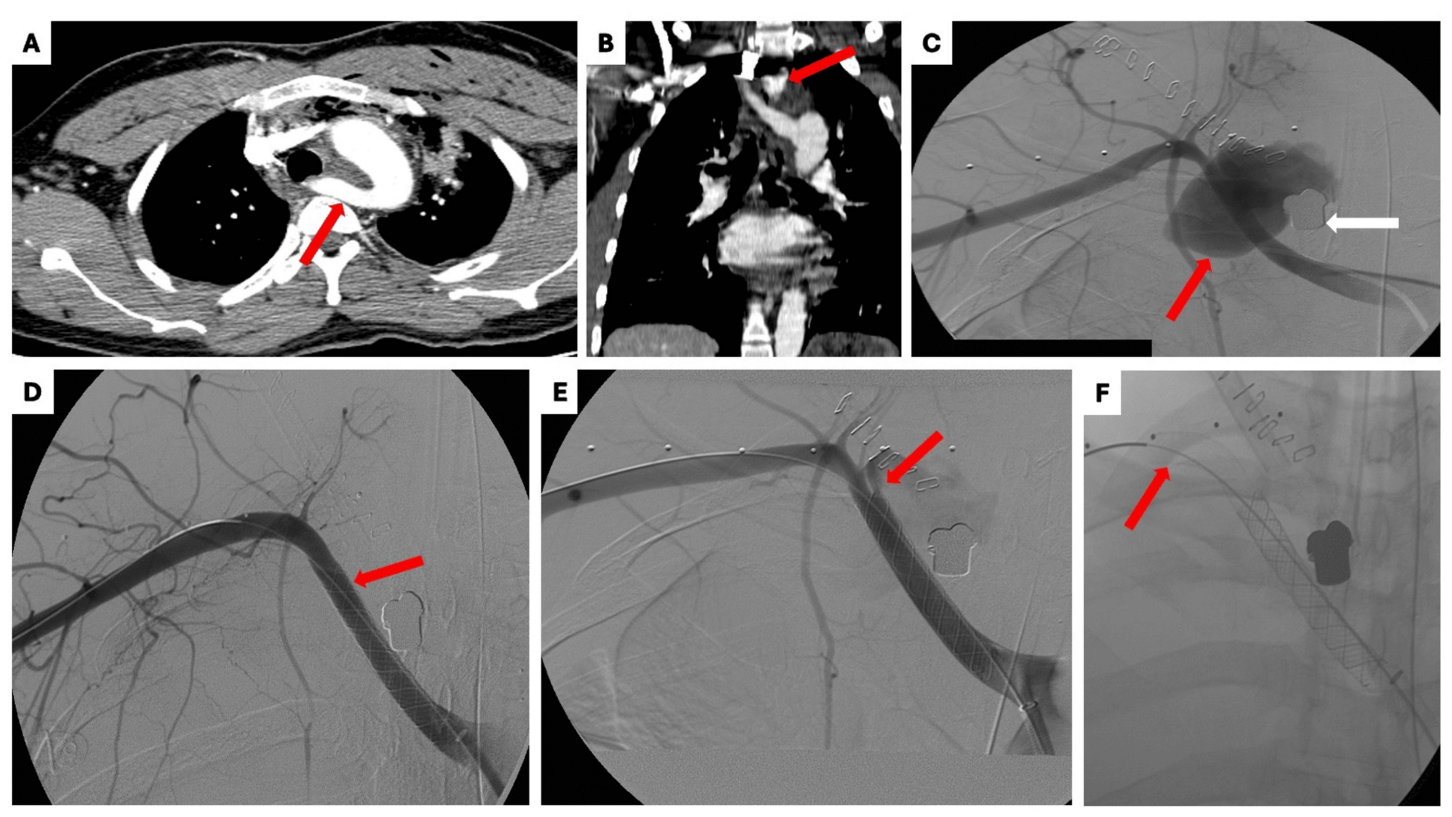
Emergent computed tomography angiogram (CTA) and postoperative imaging (A) Axial CTA with intravenous contrast at the level of great vessel origins demonstrating aberrant right subclavian artery (ARSA) (red arrow); (B) Coronal CTA of the chest demonstrating contrast-filled sac along the ARSA with irregular borders suggestive of pseudoaneurysm (red arrow); (C) Digital subtraction angiogram confirming the contrast-filled sac along the ARSA consistent with pseudoaneurysm (red arrow). Also, note the ballistic fragment adjacent to the sac (white arrow); (D) Covered stent was placed to exclude the pseudoaneurysmal sac (red arrow); (E) Immediate post-operative angiogram demonstrating type I endoleak along the distal aspect of the stent (red arrow); (F) Additional covered stent (red arrow) was placed to treat the endoleak while sacrificing the ostia for the right vertebral, thyrocervical trunk, and internal mammary arteries.

A 27-year-old male patient sustained a transmediastinal gunshot wound and presented to the emergency department hypotensive and tachycardic. Physical examination demonstrated an entrance wound in the left upper chest and cold, clammy upper extremities. Emergent computed tomography with angiography (CTA) showed a contrast-filled sac with irregular contours in contact with the ARSA, which was concerning for a pseudoaneurysm (Figure 1).

**Figure 2:**
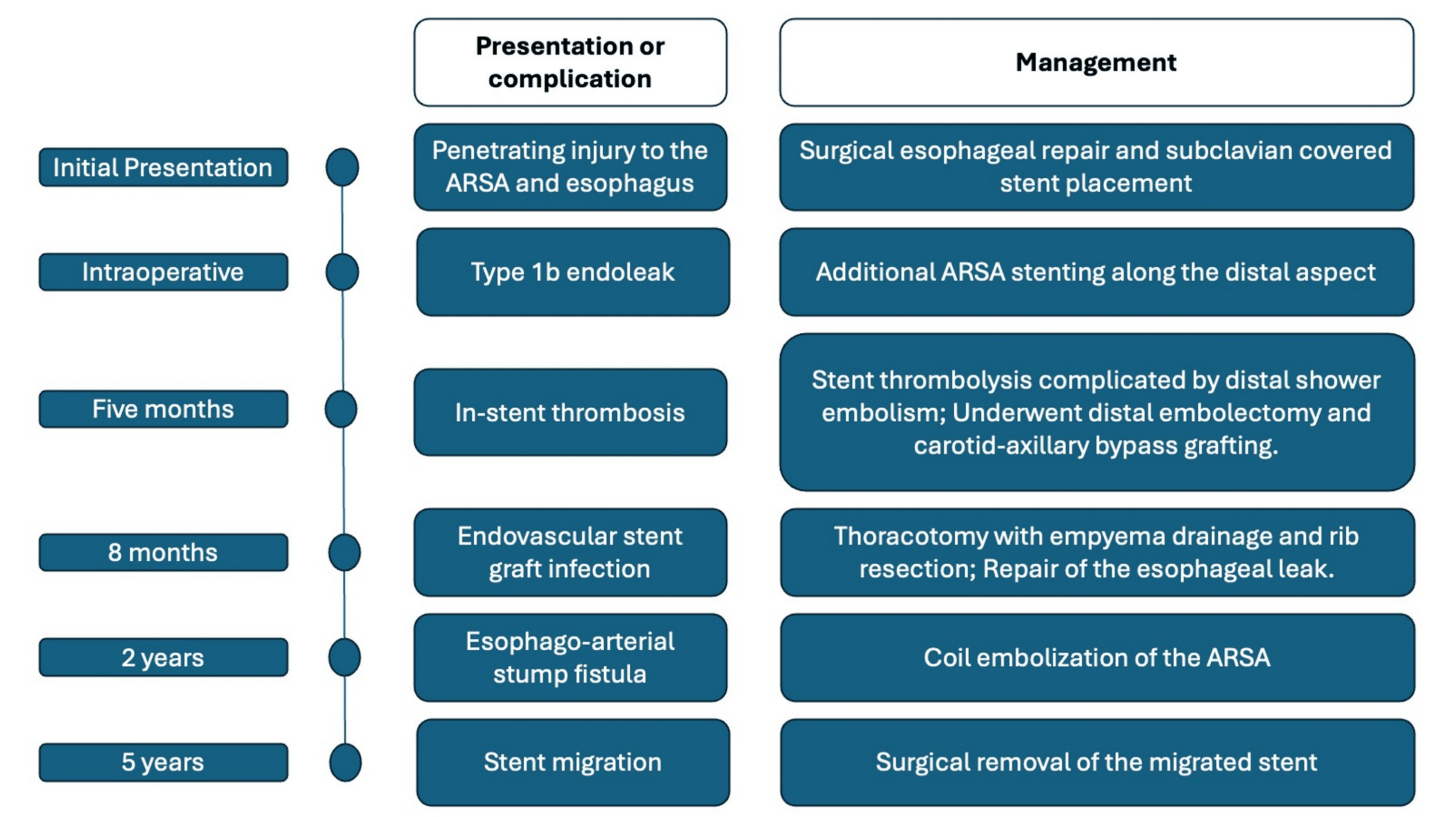
Clinical course of the patient ARSA: Aberrant Right Subclavian Artery

The patient also sustained an esophageal injury, which was repaired surgically. We will focus on the vascular complications during the patient's clinical course (Figure 2).

The ARSA pseudoaneurysm was repaired with an endovascular stent graft placement (Figure 1). However, the post deployment angiogram revealed a type 1b endoleak distal to the stent graft (Figure 1). This was managed with the placement of a second covered stent.

**Figure 3:**
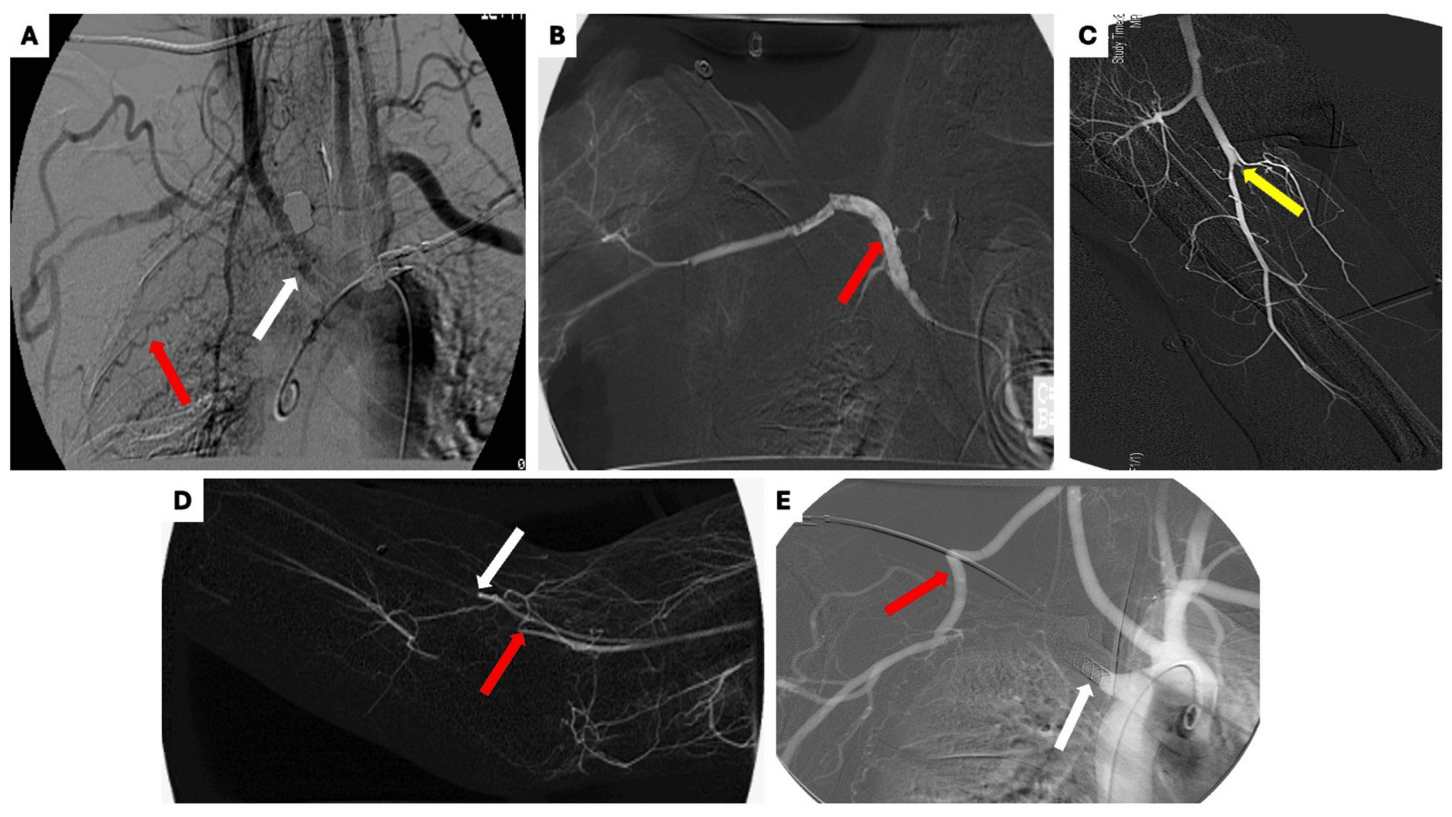
Imaging studies conducted during the clinical course (A) Digital subtraction angiography demonstrating occlusion of the proximal aspect of aberrant right subclavian artery (ARSA) (white arrow) with extensive collaterals (red arrow); (B) Pharmacologic thrombolysis was performed with alteplase which led to successful recanalization of the thrombosed ARSA (red arrow); (C & D) Thrombolysis was complicated by distal shower emboli into the proximal one-third radial (white arrow) and ulnar (red arrow) arteries as well as the proximal one-third superficial brachial artery (yellow arrow); (E) Angiogram demonstrating successful right common carotid to axillary saphenous vein bypass grafting (red arrow). Note the ARSA stump with intact stent (white arrow).

Five months after the initial presentation, the patient returned with right upper extremity tingling and digital pallor, concerning for stent stenosis or occlusion. Arteriography confirmed an occluded stent graft with collateral formation suggesting intimal hyperplasia and thrombosis (Figure 3).

Catheter-directed thrombolysis was performed with tissue plasminogen activator resulting in successful recanalization of the ARSA. However, this was complicated by distal thrombo-embolic occlusion of the proximal one-third radial, ulnar, and superficial brachial arteries. The patient subsequently underwent distal embolectomy with mechanical aspiration followed by repair of the ARSA with carotid-axillary bypass grafting. 

**Figure 4:**
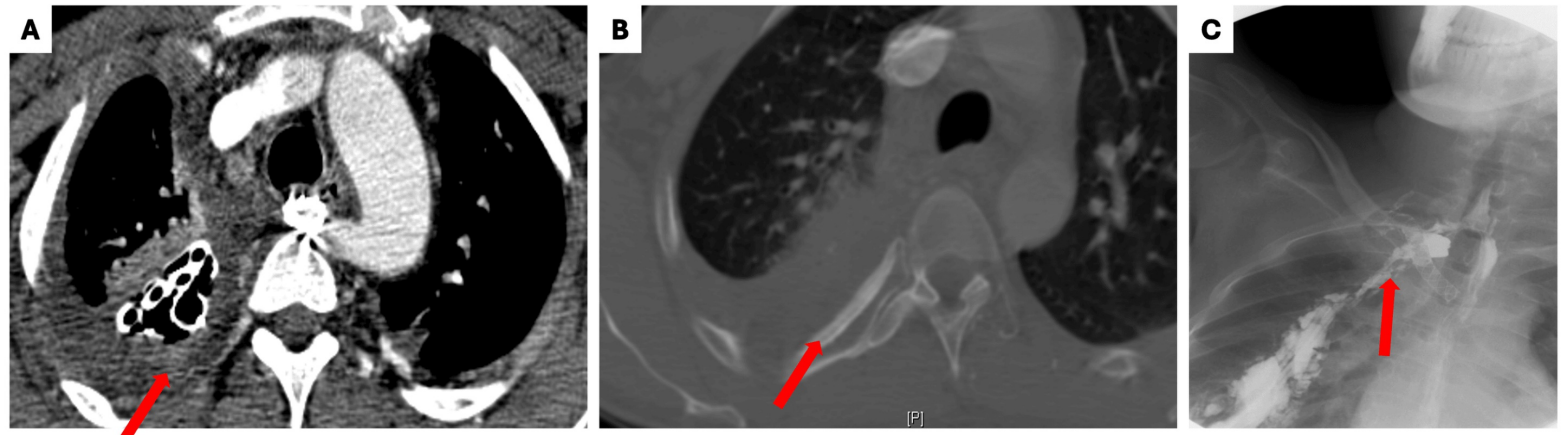
Imaging studies after eight months that indicated stent infection, periosteal reaction, and esophageal leak (A) Axial computed tomography angiogram (CTA) with intravenous contrast in soft tissue window demonstrating aberrant right subclavian artery (ARSA) stent. There is fluid collection surrounding the stent (red arrow) and also in the right pleural space, concerning for stent infection; (B) Bone window of the CTA demonstrating periosteal reaction (red arrow), indicating bone involvement leading to osteomyelitis; (C) Upper gastrointestinal fluoroscopic study demonstrating esophageal leak along the ARSA stent into the right pleural space (red arrow).

Eight months after the initial presentation, the patient presented with fever, hypotension, and leukocytosis. The CT showed mixed fluid and soft tissue density between the endovascular stent and right pleural space, raising concern for stent graft infection (Figure 4).

Periosteal reaction was also noted along the thoracic surface of the right ribs most likely representing osteomyelitis. Upper gastrointestinal fluoroscopy showed contrast extravasation at the prior site of esophageal repair, believed to be the source of stent infection. The patient underwent thoracotomy with drainage of the collection and rib resection, and the esophageal leak was surgically repaired. 

**Figure 5:**
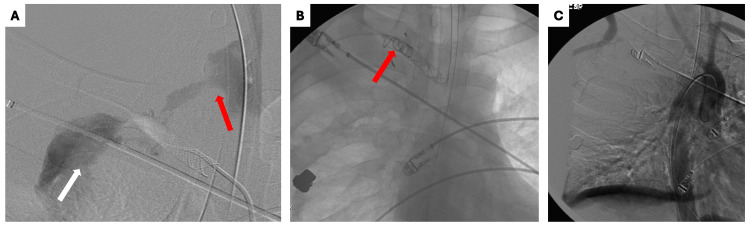
Imaging studies conducted before and after coil embolization (A) Digital subtraction angiogram demonstrating active contrast extravasation into the esophagus (red arrow) and into the right pleural space (white arrow); (B) The patient underwent coil embolization of the aberrant right subclavian artery (ARSA) (red arrow); and (C) Post-embolization aortogram demonstrated successful coil occlusion of the ARSA without active extravasation.

Two years after the initial presentation, the patient presented with mid-thoracic pain and signs of hypovolemic shock. Arteriography demonstrated active contrast extravasation from the ARSA into the esophagus and the right hemithorax (Figure 5).

Coil embolization of the ARSA was performed with completion aortogram demonstrating exclusion of the vessel and resolution of the extravasation.

**Figure 6:**
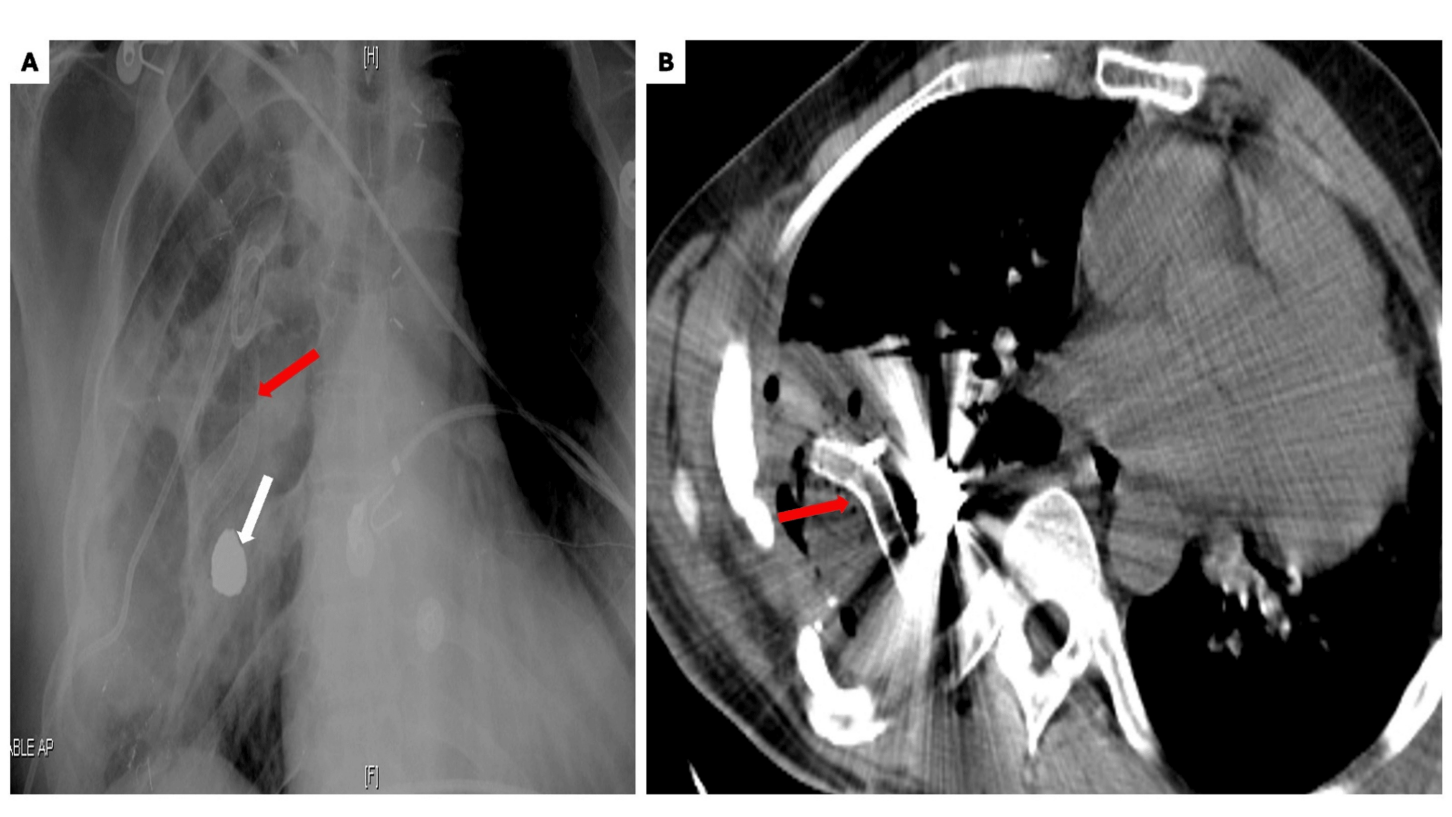
Imaging studies depicting stent migration (A) Radiograph and (B) Computed tomography demonstrating migration of the stent (red arrow) and ballistic fragment (white arrow) into the right thoracic cavity.

Five years after the initial injury, the patient presented with fever and chest pain. Emergent CT showed stent migration into the right thoracic cavity (Figure 6), likely due to chronic esophageal leak causing arterial wall erosion and eventual stent migration.

The migrated stent was surgically removed without further complications. 

## Discussion

Penetrating neck trauma has a high morbidity and mortality due to the risk of major vascular structure involvement and anatomical complexity [3]. It is associated with cervical arterial injury in 15-25% of cases. While carotid and vertebral artery injuries are more common (80% and 43% of cases, respectively) [3], subclavian artery injury is observed in approximately 1-2% of all vascular trauma cases [4,5]. The mortality rate from subclavian arterial injury ranges between 10-35% due to hemorrhagic shock [3,5].

CTA is the preferred initial imaging modality to evaluate for vascular injury. Direct imaging signs of vascular injury include intimal flap, dissection, pseudoaneurysm, partial or complete vessel occlusion, arteriovenous fistula, and vessel transection with active contrast extravasation [3]. Conventional angiography is indicated in patients with normal CTA findings and a high clinical suspicion for vascular involvement.

Pseudoaneurysm of the ARSA, as in our patient, is defined as a disruption of the arterial wall layers, leading to a contained hematoma. The incidence of subclavian artery pseudoaneurysm is 1-2% [6]. Open surgical repair, though traditional, is challenging due to anatomic complexity, high risk for hemorrhage, and brachial nerve plexus injury [6]. With the evolving minimally invasive endovascular techniques, pseudoaneurysms can be managed with covered stent placement [7]. There is a paucity of literature on the incidence and prevalence of endovascular complications related to subclavian artery stent placement [6]. Moreover, injury to the ARSA is uncommonly encountered, which further compounds the scarcity of literature.

During the intraoperative period, the stent placement in our patient was complicated by a type 1b endoleak. Although the exact prevalence of endoleaks is unknown, a systematic review described that approximately 6.1% of subclavian artery pseudoaneurysm cases develop intraoperative endoleaks [8,9].

An endoleak is defined as a persistent contrast extravasation into the pseudoaneurysm sac despite covered stent placement. Intraoperatively, our patient developed a type 1 endoleak along the distal aspect of the stent graft. Such an endoleak can be seen along the proximal or distal aspect of the stent graft and is categorized as 1a or 1b, respectively. An intraoperative endoleak can be treated with additional stenting [8]. Our patient was managed successfully with a second covered stent placement.

In-stent restenosis and occlusion of the subclavian artery is a serious complication that may lead to upper extremity ischemia. It occurs secondary to arterial wall damage resulting in neointimal hyperplasia and thrombosis. Management includes extrathoracic surgical bypass or catheter-directed thrombolysis (CDT). CDT was performed in our patient, and was complicated by distal thrombo-embolism to the radial, ulnar, and brachial arteries. This embolism was managed by embolectomy and carotid-axillary bypass grafting.

The clinical course of our patient was further complicated by endovascular stent graft infection (ESGI), most likely secondary to an esophageal leak, a complication seen in approximately 7-17% of thoracic esophageal repairs. Esophageal injury is extremely rare, is seen in 0.9-6.6% of penetrating neck trauma cases, [3] and is associated with a high mortality rate, ranging between 29 and 44% [10].

ESGI is associated with high morbidity and mortality. Major radiologic criteria for ESGI include evidence of fluid or gas accumulation around the graft on CT [11]. Other minor radiologic signs include pseudoaneurysm formation or soft tissue inflammation [11]. The infection may spread to the surrounding osseous structures resulting in osteomyelitis. Management of ESGI includes either resection of the infected graft with extra-anatomic bypass surgery or surgical debridement with an in-situ graft replacement. Our patient underwent thoracotomy with drainage of the fluid collection and resection of the rib for osteomyelitis. Additionally, the esophageal leak was surgically repaired.

Our patient later developed an esophago-arterial fistula, a rare but life-threatening condition with fewer than 30 cases reported in the literature [12]. Chiari’s triad, dysphagia or midthoracic pain, sentinel arterial hemorrhage, and exsanguination, characterizes this entity [13]. Management includes coil embolization of the ARSA or exclusion with an aortic stent graft. However, due to the proximity of the right and left subclavian arterial ostia, aortic stent grafting may result in unintentional occlusion of the left subclavian artery [14].

Eventually, the patient’s clinical course was complicated by stent migration into the thoracic cavity. As per our literature review, there are no reported cases of subclavian arterial stent migration into the thoracic cavity. We attributed this complication to a chronic esophageal leak causing arterial wall erosion. Its management required surgical removal of the migrated stent as it could be a persistent source of infection. Additionally, the esophageal leak was corrected.

## Conclusions

Penetrating injuries to the ARSA are very rare occurrences and are potentially life-threatening. When these injuries occur, they can be associated with significant morbidity and pose unique challenges in the setting of the complex anatomy in the neck and proximity to vital structures. This case illustrates the intricate and complex course of stent-related complications, requiring a multidisciplinary approach involving interventional radiology, vascular surgery, and general surgery to address the challenges involved. 

Recognizing and managing the complications associated with the condition promptly is critical, as delays in its diagnosis or treatment can lead to further complications. Among them, the most life-threatening is the development of an esophagus-arterial fistula, which is a rare and devastating condition that can lead to hemorrhage and high mortality if not promptly addressed. 
